# High-Throughput Construction of Intron-Containing Hairpin RNA Vectors for RNAi in Plants

**DOI:** 10.1371/journal.pone.0038186

**Published:** 2012-05-31

**Authors:** Pu Yan, Wentao Shen, XinZheng Gao, Xiaoying Li, Peng Zhou, Jun Duan

**Affiliations:** 1 South China Botanical Garden, Chinese Academy of Sciences, Guangzhou, China; 2 Institute of Tropical Bioscience and Biotechnology, Chinese Academy of Tropical Agricultural Science, Haikou, China; 3 Department of Basic Medical Science, Hainan Medical College, Haikou, China; 4 Graduate University of Chinese Academy of Sciences, Beijing, China; Centro de Investigación y de Estudios Avanzados del IPN, Mexico

## Abstract

With the wide use of double-stranded RNA interference (RNAi) for the analysis of gene function in plants, a high-throughput system for making hairpin RNA (hpRNA) constructs is in great demand. Here, we describe a novel restriction-ligation approach that provides a simple but efficient construction of intron-containing hpRNA (ihpRNA) vectors. The system takes advantage of the type IIs restriction enzyme BsaI and our new plant RNAi vector pRNAi-GG based on the Golden Gate (GG) cloning. This method requires only a single PCR product of the gene of interest flanked with BsaI recognition sequence, which can then be cloned into pRNAi-GG at both sense and antisense orientations simultaneously to form ihpRNA construct. The process, completed in one tube with one restriction-ligation step, produced a recombinant ihpRNA with high efficiency and zero background. We demonstrate the utility of the ihpRNA constructs generated with pRNAi-GG vector for the effective silencing of various individual endogenous and exogenous marker genes as well as two genes simultaneously. This method provides a novel and high-throughput platform for large-scale analysis of plant functional genomics.

## Introduction

After double-stranded RNA was discovered as the trigger of RNA interference (RNAi) [1], RNAi has become one of the most powerful tools for the analysis of gene function [2]–[4]. Hairpin RNA (hpRNA) constructs are commonly used to induce degradation of target genes through RNAi mechanisms [5]. In plants, intron-containing hairpin RNA (ihpRNA) with an intron as a spacer sequence shows the highest gene silencing efficiency [6]. Therefore, ihpRNA constructs have been widely used for gene silencing in plants. With the explosive release of gene sequences and genomic sequences in plants, a high-throughput and cost-efficient system for making ihpRNA constructs is in great demand.

To facilitate the generation of ihpRNA constructs, several methods have been reported. The traditional ligase-based vectors such as pHANNIBAL and pKANNIBAL were first used to generate ihpRNA constructs [6]. This method requires several rounds of restriction and ligation, and is therefore, tedious and time-consuming. Alternatively, GATEWAY cloning system-based RNAi vectors such as the pHELLSGATE series and the pIPK series have been widely used for generating ihpRNA constructs [7]–[9]. In this system, a single PCR product, from primers containing the appropriate attB1 and attB2 sites, can be simultaneously recombined into the vector to form the two arms of the hairpin. Thus, it is simpler and more rapid than the traditional ligase-based system. However, this method usually requires two cloning steps, including BP and LR reactions to generate the final ihpRNA. In addition, the reagents used in the GATEWAY system are comparatively expensive, especially for researchers in developing countries. Besides the traditional ligase-based system and the GATEWAY system, several overlap extension (OE) PCR approach were developed to construct the ihpRNA cassettes. A DA-ihpRNA method, for amplifying an ihpRNA construct directly from genomic DNA, was described by Xiao et al [10]. Our laboratory previously reported a mixed one-step OE-PCR method to generate ihpRNA constructs by assembling two inverted repeat fragments of the target genes and an intron in one tube [11]. Chen et al used a similar strategy to construct ihpRNA cassettes which were immediately inserted into the final plant expression vectors by TA cloning [12]. These PCR-based methods are not only simpler and faster than the conventional ligase-based system, but also more cost-effective than the GATEWAY system. However, they are sometimes suffer from low efficient because of the PCR suppression effect caused by the self-annealing of two inverted repeats, particularly for those target sequences that contain GC-rich regions and/or can form secondary structures. Recently, a one-step method for the generation of hpRNA constructs with 100% efficiency based on ligation independent cloning (LIC) using pRNAi-LIC vector, has been developed [13]. This method is simple and fast, as it can generate ihpRNA constructs for plant RNA silencing by one-step transformation. However, it still needs two rounds of PCR for amplifying the two inverted repeats with different adaptors and a step of restriction enzyme digestion for vector linearization before using the ligation- independent cloning.

To develop a simpler system for making RNAi constructs, we have adopted the Golden Gate (GG) cloning approach. This cloning strategy relies on the type IIs restriction enzymes, which cut outside of their recognition sequence resulting in DNA overhangs that are arbitrarily defined. This property has been used to develop protocols for efficient directional assembly of multiple DNA fragments in a single ligation reaction [14], [15]. Furthermore, with proper design of the cleavage sites, two digested fragments can be ligated to generate a product lacking the original restriction site. Thus, the two steps of digestion and ligation can be replaced by a single restriction-ligation step. Based on these properties of Golden Gate cloning, we describe here, a one-step and cost-effective method for generating ihpRNA constructs via our new plant RNAi vector pRNAi-GG. Using this system, a plant ihpRNA expression vector can be made from a single PCR product of the gene of interest by one-tube restriction-ligation reaction and one-step transformation. This method has been successfully applied to generate ihpRNA constructs for the effective silencing of various individual endogenous and exogenous marker genes as well as two genes simultaneously. Our results suggest that this novel method provides a high-throughput and reliable platform for making ihpRNA constructs, thereby, may facilitate a large-scale functional genomics analysis in plants.

## Results

### Development of a Golden Gate strategy for making ihpRNA constructs

**Figure 1:**
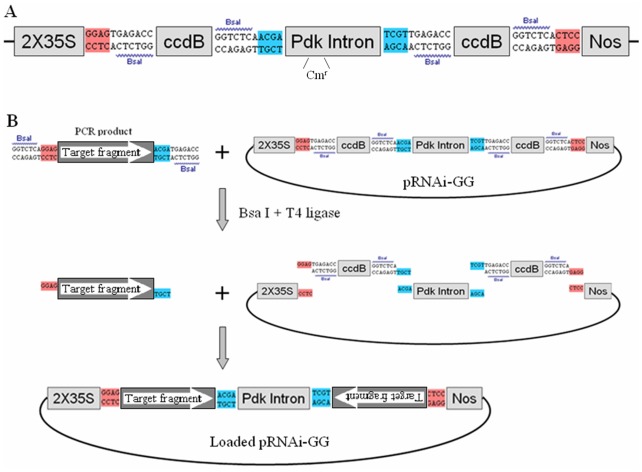
Schematic diagram of ihpRNA construction with pRNAi-GG (Golden Gate) vector. (A) The cassette of pRNAi-GG. The duplicated 35S CaMV promoter, two copies of *ccdB* gene, the Pdk intron, four BsaI recognition sites with designed adaptors (cleavage site sequences of BsaI) are cloned between the HindIII and SacI of T-DNA vector pBI121. Adaptors with the same color have the same sequences but opposite orientation. A chloramphenicol-resistant gene (Cm^r^) was contained in the Pdk intron. (B) One-step construction of ihpRNA. The target fragment of the gene of interest is PCR amplified using gene-specific primers carrying BsaI sites and adaptors complementary to the appropriate sequences on the vector. The purified PCR product is mixed, in one tube, with pRNAi-GG vector, BsaI enzyme and T4 ligase for a one-step restriction-ligation reaction.

To adopt a Golden Gate strategy for the rapid and high-throughput cloning of the intron-containing inverted repeat inserts into the vectors, we developed a new plant T-DNA RNAi vector, pRNAi-GG. This vector consists of a *ccdB* gene - Pdk intron - *ccdB* gene cassette between the duplicated CaMV 35S promoter and Nos terminator (Fig. 1A). One BsaI recognition site, in the original *ccdB* gene, was eliminated by site-specific mutation without changing the coded amino acid sequence. Two BsaI sites flank each *ccdB* gene and are positioned such that the recognition sites are eliminated from the vector after proper digestion and ligation of products. The cleavage site sequences of BsaI on the left of the first *ccdB* gene are the same to that on the right of the second *ccdB*, but with different orientation, and the same to the other two BsaI sites. Thus, a single PCR product can replace the two *ccdB* genes simultaneously at both sense and antisense orientations to form the two arms of the hairpin (Fig. 1B). To make the ihpRNA construct, the target region of the gene of interest is amplified with BsaI recognition sites flanked at both ends, and the cleavage site sequences (adaptor for pRNAi-GG) are complementary to the appropriate sequences on the vector. The universal form for primer design is 5′- protective bases - BsaI - adaptor for pRNAi-GG - gene specific sequence-3′. Incubation of the purified PCR product and pRNAi-GG in the presence of BsaI enzyme and T4 ligase generates the desired vector, which is stable due to the absence of the original BsaI sites (Fig. 1B). The mixture can then be transformed directly into standard E. coli strains such as DH5α and TOP10 (but not in DB3.1), and only recombinants containing both arms would be recovered. Since pRNAi-GG is a binary vector, the recombinant pRNAi-GG constructs can be transformed directly into Agrobacterium for plant transformation.

### Fast and efficient cloning of plant ihpRNA constructs with pRNAi-GG

**Figure 2:**
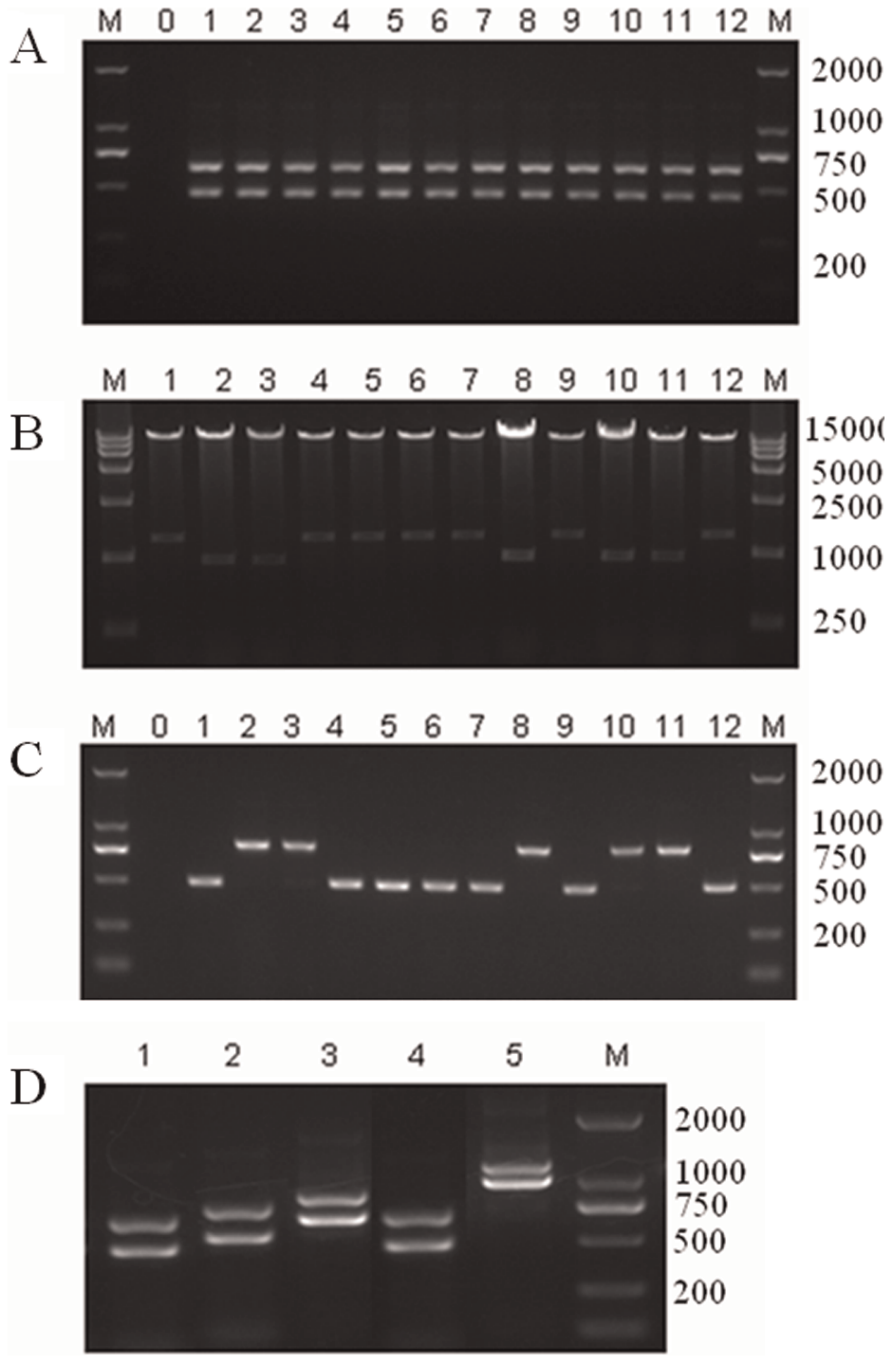
Zero-background cloning of intron-containing hairpin RNA (ihpRNA) constructs. (A) Colony PCR of pRNAi-GFP using primes P21, P22 and P12, which can amplify the two arms simultaneously with a difference of 267 bp in length. (B) Double digestion identification of pRNAi-GFP with BamHI and SacI. The two different sizes of inserts are due to the different orientation of the intron. (C) Identification of the intron orientation of pRNAi-GFP by colony PCR using primes P21, P24 and P25. Lanes 1–12 in (A–C) represent 12 independent colonies, Lane 0 is the negative control and M is DNA marker. (D) Colony PCR results of 5 ihpRNA constructs, lines 1–5, for silencing GFP, GUS, NbPDS, GFP with an internal BsaI site and GFP/NbPDS, respectively. The lengths of the inverted arms are 337 bp, 460 bp, 530 bp, 368 bp and 867 bp, respectively. All constructs gave the predicted bands.

To test the efficiency of our new plant RNAi vector pRNAi-GG for making ihpRNA constructs, a target region with 337 bp in the marker gene *GFP* (GenBank access number U87973) was amplified, and mixed with pRNAi-GG in the presence of BsaI enzyme and T4 ligase. To the efficiency of cloning at different restriction-ligation time, the mixture was incubated at 37°C for 0.5–2 h or for 20–35 cycles (2 min 37°C+5 min 16°C). After 5 minutes at 80°C for heat inactivation, this mixture was transformed into E. coli DH5α cells and transformants were selected on LB plates containing both kanamycin and chloramphenicol. As shown in Table 1, recombinant colonies increased from 0.5 to 2 h or form 20 to 35 cycles. The efficiency of cloning at 37°C for 2 h is higher than 20 cycles, but a little lower than 35 cycles. Considering the time efficiency, incubated at 37°C for 2 h is commended to be the optimal condition. For each transformation, 12 clones (when less than 12, pick all) were identified by PCR using vector specific primes P21 and P22, and insert reverse primer P12 (Fig. S1, Table S1), which can amplify the two arms simultaneously with a difference of 267 bp in length. All the clones showed the expected bands by agarose gel electrophoresis, as part of the results was shown in Fig. 2A. 12 clones were also identified by restriction enzymes digestion using BamHI and SacI (Fig. S1). As shown in Fig. 2B, all of the colonies contain the correct inserts, and the two different sizes of inserts in digestion are due to the different orientation of the intron. The intron orientation can also be easily identified by colony PCR using a combination of two intron specific primers P24 and P25 and one vector specific primer P21 (Table S1, Fig. 2C). The vector–insert junctions, of six colonies, were sequenced using P22 and P23 primers and all of them contained the correct sequences as expected.

**Table 1:** Efficiency of cloning of pRNAi-GG at different restriction-ligation times.

Construct	neg	0.5 h 37°C	1 h 37°C	2 h 37°C	20 cycles	35 cycles	50 cycles
pRNAi-GFP	0	16	24	37	35	48	NT
pRNAi-GFP	0	9	22	45	26	43	NT
pRNAi-GFP/PDS	0	NT	NT	2	2	13	27
pRNAi-GFP/PDS	0	NT	NT	5	3	8	16

The number of recombinant colonies for each transformation was counted. Restriction-ligation was performed with continuous incubation at 37°C or for 20–50 cycles (2 min 37°C+5 min16°C). Cloning for pRNAi-GFP and pRNAi-GFP/PDS were performed in duplicate. The negative control was performed without BsaI enzyme. NT, not tested.

For each transformation, 12 clones (when less than 12, pick all) were identified by PCR using vector specific primes P21 and P22, and insert reverse primer P12 (for pRNAi-GFP) or P16 (for pRNAi-GFP/PDS). All the clones showed the expected bands, as part of the results was shown in Fig. 2A.

In pRNAi-GG, a chloramphenicol-resistant gene was inserted into the Pdk intron to allow for selecting the presence of the intron (Fig. 1A). To test the importance of the chloramphenicol-resistant gene, in recovering the correct recombination products, we compared the recombinant plasmids in the presence and absence of chloramphenicol. Over 50 chloramphenicol-resistant clones were tested by PCR, and all was found to have the correct structure. While in the absence of chloramphenicol, 10–20% of the clones were found to don't have the intron. Sequences from three illegitimate recombinants showed products of recombinant of the vector itself at the position of two Nos terminators (data not shown). Therefore, we conclude that a selectable marker in the intron is necessary for the generation of ihpRNA constructs when using this system.

In order to demonstrate that the use of vector pRNAi-GG is reproducible and reliable to make ihpRNA constructs with the Golden Gate method, we also successfully generated other recombinant pRNAi-GG constructs, including the target regions from the *N. benthamiana* PDS gene (*NbPDS*) (GenBank access number EU165355) and marker gene *GUS* (GenBank access number AY292368), as well as another target region from *GFP* with an internal BsaI site (Fig. 2D). To bypass the internal BsaI site problem, a two step ligation was employed. After the first step of restriction-ligation for 2 h, the enzymes were heat inactivated, and then fresh ligase was added to the mix for the second ligation for 1 h.

### Silencing of exogenous marker genes and endogenous NbPDS by agroinfiltration

**Figure 3:**
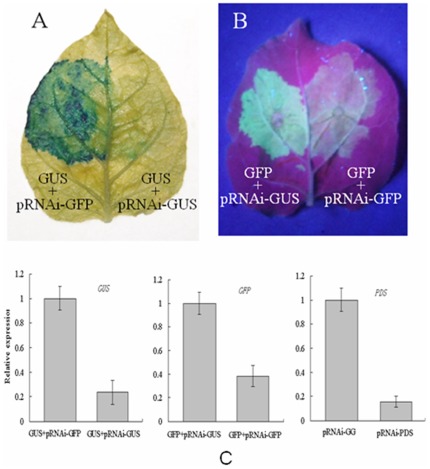
Silencing of two marker genes GUS and GFP and endogenous NbPDS by transient expression of the ihpRNA constructs. (A) Silencing effect of GUS ihpRNA on transient expression of GUS gene. Agrobacterium cultures containing pBIN19-GUS were mixed with Agrobacterium containing pRNAi-GFP (left) or pRNAi-GUS (right), and infiltrated into different parts of the same leaves of *N. benthamiana* plants. (B) Silencing effect of green ﬂuorescent protein (GFP) ihpRNA on transient expression of GFP gene. Agrobacterium cultures containing pBIN19-GFP were mixed with Agrobacterium containing pRNAi-GUS (left) or pRNAi-GFP (right), and infiltrated into different parts of the same leaves of *Nicotiana benthamiana* plants. (C) Real time PCR was performed to analyze the silencing effect of GUS, GFP and NbPDS genes. Error bars represent standard deviations (SD) of three independent experiments.

Coinfiltration has been widely used to determine the functionality of RNAi constructs for silencing of marker genes. In order to demonstrate that the ihpRNA constructs (described above) can be used for gene silencing, we first tested the ability of pRNAi-GUS and pRNAi-GFP for gene silencing by Agrobacterium-mediated transient expression. Agrobacterium cultures, each containing pBIN19-GUS and pBIN19-GFP were mixed with Agrobacterium cultures, each containing the pRNAi-GUS or pRNAi-GFP (for control), and pRNAi-GFP or pRNAi-GUS (for control), respectively. The mixed Agrobacterium cultures were infiltrated into different parts of the same leaves of *N. benthamiana* plants. The infiltration areas for control gave a strong blue GUS-staining signal (Fig. 3A) or green ﬂuorescence (Fig. 3B) at 3 d post-agroinfiltration. However, the infiltration areas for gene silencing gave much weaker signals. To confirm the knock-down of the test marker genes at the molecular level, we performed real time PCR. The results showed that GUS and GFP mRNA levels were reduced to 24% and 36%, respectively, compared to the control samples (Fig. 3C). In addition to the exogenous marker genes, the mRNA level of *NbPDS* was also reduced by the infiltration of Agrobacterium cultures containing pRNAi-PDS. The result of real time PCR showed that the expression of *NbPD*S at 3 d post-agroinfiltration was reduced to 17%, compared to the control samples (Fig. 3C). However, the typical phenotype of *PDS* silencing, such as the photo-bleaching in VIGS (virus-induced gene silencing), was not observed in the infiltration areas even 15 days post infiltration. The reason may be that the RNA interference mediated by agroinfiltration is transient and reacts on small area of the plant, which couldn't reach the level to change the phenotype. Usually, Agrobacterium-mediated transient expression of a transgene achieves highest level in 2–3 days following argoinfiltration, after which the expression level rapidly decreases [16].

### The effect of intron orientation of the recombinant pRNAi-GG on silencing efficiency

**Figure 4:**
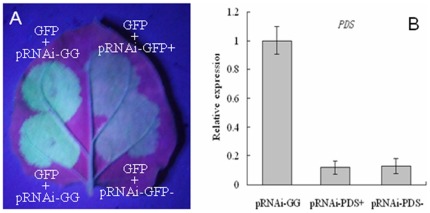
The effect of intron orientation of recombinant pRNAi-GG on silencing efficiency. (A) Silencing of *GFP* by pRNAi-GFP+ and pRNAi-GFP−. (B) Real time PCR was performed to analyze the silencing of *NbPDS* by pRNAi-PDS+ and pRNAi-PDS−. + and − indicate the sense and antisense orientations of intron of recombinant pRNAi-GG, respectively. Empty vector pRNAi-GG was used as a control. Error bars indicate SD of three independent experiments.

Due to the same cleavage sites of BsaI at both sides of Pdk intron in the pRNAi-GG vector, we observed that the recombinant ihpRNA vectors included plasmids in which the intron retained its forward or reversed orientation with respect to the promoter. The intron orientation can be easily identified either by colony PCR using a combination of two intron specific primers P24 and P25 and one vector specific primer P21 (Table S1, Fig. 2D) or by restriction enzymes digestion using BamHI and SacI (Fig. 2C). The position of the primers and the restriction sites on pRNAi-GG were showed in Fig. S1. To test the effect of intron orientation of the recombinant pRNAi-GG on gene silencing, pRNAi-GFP and pRNAi-PDS with introns in either sense or antisense orientations were selected and tested for transient expression. As shown in Fig. 4, no obvious difference in silencing efficiency was observed between the recombinant pRNAi-GG with different intron orientations. Therefore, the selection of intron orientation is not critical, and if necessary, it can be easily identified by colony PCR or restriction enzyme digestion.

### Simultaneous silencing of *GFP* and *NbPDS*

**Figure 5:**
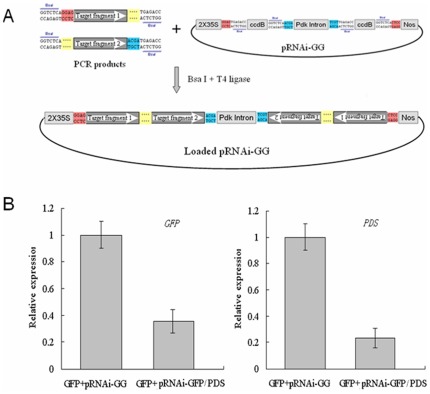
Simultaneous silencing of two genes. (A) Schematic diagram of hybrid ihpRNA construction with pRNAi-GG vector. Two PCR products representing genes of interest can be cloned into pRNAi-GG simultaneously in a single restriction-ligation reaction. (B) Real time PCR was performed to analyze the simultaneous silencing of *GFP* and *NbPDS* by transient expression using pRNAi-GFP/PDS. Error bars represent SD of three independent experiments.

Considering the extremely high efficiency of Golden Gate cloning, it was logical to test whether multiple fragments could be inserted to pRNAi-GG. For this purpose, we developed a strategy to construct an ihpRNA targeting two distinct genes in a single cloning step (Fig. 5A). Both of the inserts are flanked with BsaI sites whose cleavage site sequences are designed such that the two inserts can be ligated after the digestion. The efficiency of cloning at different restriction-ligation time was tested, and the results showed the optimal condition is incubation for 50 cycles of 2 minutes at 37°C and 5 minutes at 16°C (Table 1). By employing this strategy, an ihpRNA, namely pRNAi-GFP/PDS, targeting both *GFP* and *NbPDS* was successfully constructed (Fig. 2D). Agrobacterium cultures containing the GFP and the pRNAi-GFP/PDS were coinfiltrated into the leaves of *N. benthamiana* plants. The simultaneous silencing of *GFP* and *NbPDS* was observed 3 d post-agroinfiltration (Fig. 5B).

## Discussion

RNA silencing induced by hpRNA is one of the most powerful and popular tools in reverse genetics. ihpRNA constructs have been widely used for the analysis of gene function in plants. With the explosive release of gene sequences and whole genome sequences in plants, how to rapidly determine the gene function on a large scale is a daunting challenge. In this study, we developed a novel strategy and a new plant RNAi vector, pRNAi-GG, for making ihpRNA constructs rapidly with only one restriction-ligation cloning step. We have demonstrated that the ihpRNA constructs, generated with this RNAi vector based on Golden Gate cloning method, can efficiently induce suppression of target genes. This novel method and our plant RNAi vector provide a high-throughput platform for the large-scale plant functional genomic analyses.

The method we described has many advantages over both the traditional ligase-based and GATEWAY systems. First, ihpRNA constructs can be produced in one tube with one restriction-ligation step. The cloning process of ihpRNA constructs can be completed within several hours. Second, our method requires only a single primer pair and ordinary reagents which are relatively cost-savings. Third, with both the *ccdB* and chloramphenicol-resistant genes, our new vector pRNAi-GG can be used to make ihpRNA constructs with zero-background cloning efficiency. Fourth, pRNAi-GG is a binary vector; thereby, allowing for the recombinant pRNAi-GG constructs to be directly transformed into Agrobacterium for plant transformation. Taken together, these advantages greatly facilitate and enhance the high-throughput applications for large scale gene functional analysis.

Recently, a one-step method for the generation of hpRNA constructs with 100% efficiency based on ligation independent cloning (LIC) using pRNAi-LIC vector, has been developed [13]. This method has almost all of the advantages described above. However, it still requires two rounds of PCR for amplifying the two inverted repeats and a step of restriction enzyme digestion for vector linearization before cloning. On the other hand, our method only calls for a single PCR product, which can be immediately cloned into pRNAi-GG without any other requirements. Therefore, our method is simpler and faster. At least, it is a good alternative system for making RNAi constructs with high-throughput.

Due to the same cleavage sites of BsaI on both sides of Pdk intron in the pRNAi-GG vector, we observed that the recombinant ihpRNA vectors included plasmids in which the intron either was reversed or retained its forward orientation with respect to the promoter. Similar result was also observed in the pHELLSGATE vector based on the GATEWAY system [7], [17]. The reverse orientation of intron in the recombinant ihpRNA was usually considered to have negative effect in silencing efficiency, so in some RNAi vectors the spacer fragments were designed to consist of two introns in opposite orientations to ensure one of the introns is in the forward orientation [7]. However, no data was reported for the effect of intron orientation on gene silencing. We compared the silencing efficiency of ihpRNA vectors with both sense and antisense of intron orientations, and found that the orientation of intron has no obvious effect on silencing efficiency. Therefore, the selection of intron orientation is not critical, and if necessary, it can be easily identified by colony PCR or restriction enzyme digestion.

Our method is based on type IIs restriction enzyme BsaI. Therefore, one potential limitation of this method might come from the occasional presence of one to several internal BsaI site(s) in the gene of interest. In order to solve this problem, we performed the restriction-ligation for 2 h, followed by heat inactivation of the enzymes, and then another 1 h ligation by adding fresh ligase. As a result, an ihpRNA containing an internal BsaI restriction site was successfully constructed (Fig. 2D). We found this to be extremely efficient since the restriction-ligation, in the first step, stably ligates the insert fragments at the ends of the vector, and the second ligation only needs to religate the overhangs at the internal BsaI sites [15]. Since the BsaI restriction site has a 6-base pair recognition sequence, it is expected to be present on average every four kb of the genome. Since the stem length of ihpRNA is usually around 500 bp, the probability of housing more than one BsaI site is very unlikely. Thus, the occasional presence of internal BsaI site will not limit the application of this method.

Golden Gate cloning is based on the use of type IIs restriction enzymes and allows the assembly of multiple DNA fragments in a restriction-ligation reaction. Several protocols based on Golden Gate cloning have been reported for DNA Shuffling [14], assembly of genetic modules [18], [19], long-length DNA [20] and transcription activator-like (TAL) effectors [21]–[24]. In this study, we adopted Golden Gate cloning to make ihpRNA constructs for high-throughput. In our method, the recombinant could be immediately generated between PCR products and the destination plasmid, rather than the entry and destination plasmids in the original Golden Gate cloning. This means that the subcloning of PCR product is unnecessary, which further enhances the high-throughput construction of ihpRNA.

In summary, we have developed a simple, rapid but reliable method for making ihpRNA constructs. This method proves to be promising for a wide application for the large-scale analysis of plant functional genomics.

## Methods

### Plant and plasmid materials

Wild-type *Nicotiana benthamiana* was used for the analysis of transient silencing. pBIN19-GUS and pBIN19-GFP are pBIN19-based and used for the expression of GUS and GFP, respectively. Both plasmids were kindly provided by Dr. Mestre [25]. pBI121 is a binary vector for plant transformation [26].

### Plant RNAi vector pRNAi-GG Construction

pRNAi-GG vector was generated using the T-DNA vector pBI121 as the skeleton. The duplicated CaMV 35S promoter was amplified using pSATN-cEYFP-C1 [27] as template. Two copies of *ccdB* gene and the Pdk intron-containing chloramphenicol resistant gene were PCR amplified using pRNAi-LIC [13] as template. The BsaI site in the original *ccdB* gene was removed by overlap PCR method without changing its amino acid sequence, and both of the modified *ccdB* genes are flanked with BsaI sites. Sequences of these primers are listed in Table S1. All four PCR products were cloned into T-vector pMD-18T and then digested with HindIII and ApaI, ApaI and SalI, SalI and XbaI, XbaI and SacI, respectively. Finally, the four digested fragments were ligated into HindIII- and SacI-digested pBI121, in one tube, to generate pRNAi-GG. pRNAi-GG was used to generate all ihpRNA constructs for the silencing of genes used in this sudy, and maintained in E. coli strain DB3.1, in which the *ccdB* gene is not lethal. The full-length sequence and detailed annotation of pRNAi-GG was deposited in GenBank (access number JQ085427).

### Golden Gate cloning with pRNAi-GG for making ihpRNA constructs

Only a single PCR product is required to make the ihpRNA construct for silencing the gene of interest with pRNAi-GG vector by Golden Gate cloning. The product is amplified by the oligo pair: 5′- acca ggtctc aggag - gene specific forward primer-3′ and 5′-acca ggtctc atcgt- gene specific reverse primer-3′ (5′- protective bases - BsaI - adaptor for pRNAi-GG - gene specific sequence-3′). The Golden Gate reaction for making ihpRNA constructs is set up by pipetting into a tube 50 ng purified PCR product, 200 ng pRNAi-GG vector, 5 units BsaI enzyme (NEB) and 10 units T4 DNA ligase (Promega, high concentration ligase - 20 u/µl) in a total volume of 10 µl in 1× ligation buffer (Promega). The restriction-ligation is incubated at 37°C for 2 hours, followed by incubation for 5 minutes at 50°C (final digestion) and then 5 minutes at 80°C (heat inactivation). Then 5–10 µl of the mixture was transformed into E. coli DH5α competent Cells and plated on LB medium containing 25 mg/L kanamycin and 5 mg/L chloramphenicol to select the recombinants. The ihpRNA constructs for silencing GUS, GFP, and NbPDS genes were generated by this restriction-ligation reactions and named as pRNAi-GUS, pRNAi-GFP and pRNAi-NbPDS, respectively. These constructs were confirmed by PCR and DNA sequencing. Sequences of these primers are listed in Table S1.

### Transient expression by agroinfiltration

Agroinfiltration was performed following previously reported procedures with a slight modification [28]. *Nicotiana benthamiana* plants were grown in a growth chamber under standard conditions at 25°C under 16-h-light/8-h-dark cycle. All ihpRNA constructs were transformed into Agrobacterium strain GV3101. A single colony of each transformed Agrobacterium was used to inoculate 5 mL YEP medium (Bacto-Trypton, 10 g/l; yeast extract, 10 g/l; NaCl, 5 g/l; pH 7.0) supplemented with 100 mg/l Rifampicinand and 50 mg/l kanamycin. Bacteria were grown overnight to obtain an OD600 of 1.0–1.5 at 28°C, shaking at 200 rpm. The cultures were pelleted by centrifugation at 2,000 g for 5 min and cells were diluted with infiltration buffer (50 mM MES pH 5.6, 10 mM MgCl2, and 100 µM acetosyringone) to a final OD600 of 0.1–0.3, and then incubated for 1–2 h at 25°C in the dark before agroinfiltration of *N. benthamiana* plants using a 1-ml needleless syringe.

### GUS and fluorescence assays

GUS assay was performed as per previously described procedures with a slight modification [29]. The infiltrated *Nicotiana benthamiana* leaves were detached 3 d post-agroinfiltration and put into GUS staining buffer (50 mM phosphate buffer (pH 7.0), 10 mM Na_2_EDTA, 5 mM K3Fe(CN)6, 5 mM K4Fe(CN)6, 0.1% X-Gluc). The leaves were then incubated for 2–10 h at 37°C in the dark, with continuous gentle shaking. When blue color appeared on the leaves, the leaves were washed in ddH_2_O and then placed into 75% ethanol to fix and remove chlorophyll. Green fluorescence protein was detected using a long-wavelength UV lamp (Black Ray model B 100A; UV products; Upland, CA, USA) and photographs were taken using Cannon EOS 550D digital camera.

### RNA isolation and real-time PCR analysis

Real-time PCR was used to examine the level of RNA accumulation. Total RNA was isolated from leaf tissues, by using RNAprep pure plant kit (Tiangen, Beijing, China), and treated with RNase-free DNase I to remove DNA contamination. First-strand cDNA was synthesized from 500 ng of total RNA using SYBR PrimeScript RT-PCR Kit II (Takara, Dalian, China). Real-time PCR was performed on Stratagene MX3000P with three technical and three biological replicates. Sequences of these primers are listed in Table S1. The relative expressions of the genes of interest were calculated using the formula 2^−ΔΔCT^ [30], by normalizing to the expression levels of Actin of *N. benthamiana* (GenBank access number AY594294).

### Ethics Statement

No specific permits were required for the described field studies. No specific permissions were required for these locations/activities. The location is not privately-owned or protected in any way. The field studies did not involve endangered or protected species.

## Supporting Information

Figure S1 The position of the primers and the restriction sites on pRNAi-GG used for the identification of recombinant pRNAi-GG or intron orientation. P21, P22 and insert reverse primer were designed to identify the recombinant pRNAi-GG, by amplifying two arms simultaneously with a difference of 267 bp in length. P21, P24 and P25 were used to identify the intron orientation. The PCR product of the recombinant pRNAi-GG with sense orientation of intron is 309 bp longer than that of the recombinant pRNAi-GG with antisense orientation of intron. BamHI and SacI can also be used to identify the intron orientation. The digested inserts of the recombinant pRNAi-GG with sense orientation of intron is 405 bp shorter than that of the recombinant pRNAi-GG with antisense orientation of intron.
(TIF)

Table S1 Primer sequences used in this study.
(DOC)
