# Supplementary figures and images for: High-Throughput Construction of Intron-Containing Hairpin RNA Vectors for RNAi in Plants
Source: PLoS One. 2012 May 31;7(5):e38186. doi: 10.1371/journal.pone.0038186 (PMC3364983; doi:10.1371/journal.pone.0038186)

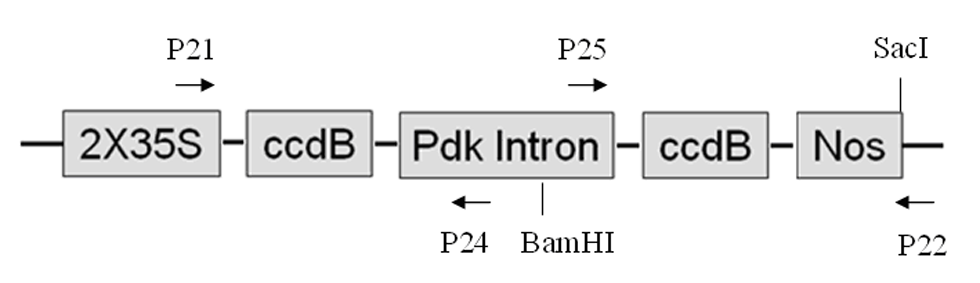

Supplement: Figure S1 — The position of the primers and the restriction sites on pRNAi-GG used for the identification of recombinant pRNAi-GG or intron orientation. P21, P22 and insert reverse primer were designed to identify the recombinant pRNAi-GG, by amplifying two arms simultaneously with a difference of 267 bp in length. P21, P24 and P25 were used to identify the intron orientation. The PCR product of the recombinant pRNAi-GG with sense orientation of intron is 309 bp longer than that of the recombinant pRNAi-GG with antisense orientation of intron. BamHI and SacI can also be used to identify the intron orientation. The digested inserts of the recombinant pRNAi-GG with sense orientation of intron is 405 bp shorter than that of the recombinant pRNAi-GG with antisense orientation of intron. (TIF) [file pone.0038186.s001.tif]
