# Supplementary material for: High-Throughput Construction of Intron-Containing Hairpin RNA Vectors for RNAi in Plants
Source: PLoS One. 2012 May 31;7(5):e38186. doi: 10.1371/journal.pone.0038186 (PMC3364983; doi:10.1371/journal.pone.0038186)
Supplement: Table S1 — Primer sequences used in this study. (DOC) [file pone.0038186.s002.doc]

Table S1. Primer sequences used in this study

| Primer | Sequence (5′-3′) | Description |
| --- | --- | --- |
| P1 | tcc aagctt tcaacatgtggagcacgacac | For 2×35S PCR |
| P2 | agc gggccc tggctatcgttcgtaaatggt | For 2×35S PCR |
| P3 | cgg gggccc ggagtgagacc aattctcgactaagttggcag | For the first ccdB PCR |
| P4 | gca gtcgac ctgtgtataagggagcctgac | For the first ccdB PCR |
| P5 | tcg tctaga tcgttgagacc aattctcgactaagttggcag | For the second ccdB PCR |
| P6 | tcc gagctc ggagtgagacc ctgtgtataagggagcctgac | For the second ccdB PCR |
| P7 | gca gtcgac ggtctcaacga gcccttggtaaggaaataattattttc | For PDK intron PCR |
| P8 | ctg tctaga ccaactgtaatcaatccaaatg | For PDK intron PCR |
| P9 | agtgtgccggtatccgttatcg | For ccdB mutation |
| P10 | cgataacggataccggcacact | For ccdB mutation |
| P11 | acca ggtctcaggag gacgggaactacaagacacg | For pRNAi-GFP |
| P12 | acca ggtctcatcgt tcttttcgttgggatctttcg | For pRNAi-GFP |
| P13 | acca ggtctcaggag agcgttgaactgcgtgat | For pRNAi-GUS |
| P14 | acca ggtctcatcgt cgaaaccaatgcctaaag | For pRNAi-GUS |
| P15 | acca ggtctcaggag tgcttttgtgtttgccactc | For pRNAi-PDS |
| P16 | acca ggtctcatcgt caccttccattgaagccaag | For pRNAi-PDS |
| P17 | acca ggtctcaggag gacgggaactacaagacacg | For pRNAi-GFP with an internal BsaI site |
| P18 | acca ggtctcatcgt gttacaaactcaagaaggacc | For pRNAi-GFP with an internal BsaI site |
| P19 | acca ggtctca tcttttcgttgggatctttcg | For pRNAi-GFP/PDS |
| P20 | acca ggtctcgaaga tgcttttgtgtttgccactc | For pRNAi-GFP/PDS |
| P21 | accatttacgaacgatagcc | For recombinants identification |
| P22 | gtaaaacgacggccagtg | For recombinants identification and sequencing |
| P23 | cgaatctcaagcaatcaagc | For recombinants sequencing |
| P24 | cattttagcttccttagctcc | For intron orientation identification |
| P25 | catttggattgattacagttgg | For intron orientation identification |
| P26 | gggtgaaggtgatgcaacat | For GFP RT-PCR |
| P27 | aagaagtcgtgccgcttcat | For GFP RT-PCR |
| P28 | aatccatcgcagcgtaatgc | For GUS RT-PCR |
| P29 | accacctgccagtcaacaga | For GUS RT-PCR |
| P30 | cccgaagattgacaaaggac | For PDS RT-PCR |
| P31 | cgtgaggaagtacgaaacga | For PDS RT-PCR |
| P32 | taacccaaaggctaatcgtg | For NbActin RT-PCR |
| P33 | tccaacacaataccggtggt | For NbActin RT-PCR |
